# Early macrophage infiltrates impair pancreatic cancer cell growth by TNF-α secretion

**DOI:** 10.1186/s12885-020-07697-1

**Published:** 2020-12-02

**Authors:** Cansu Tekin, Hella L. Aberson, Maarten F. Bijlsma, C. Arnold Spek

**Affiliations:** 1 Amsterdam University Medical Centers, University of Amsterdam, Center of Experimental and Molecular Medicine, Meibergdreef 9, 1105AZ Amsterdam, The Netherlands; 2 Amsterdam UMC, University of Amsterdam, Laboratory for Experimental Oncology and Radiobiology, Cancer Center Amsterdam, Amsterdam, The Netherlands; 3 Oncode Institute, Amsterdam, The Netherlands

**Keywords:** Pancreatic cancer, PDAC, Macrophages, Tumor-associated macrophages, TNF-α, apoptosis

## Abstract

**Background:**

Pancreatic ductal adenocarcinoma (PDAC) is a grim disease with high mortality rates. Increased macrophage influx in PDAC is a common hallmark and associated with poor prognosis. Macrophages have high cellular plasticity, which can differentiate into both anti- and pro-tumorigenic properties. Here, we investigated how naïve (M0) macrophages differ from other macrophages in their anti-tumorigenic activities.

**Methods:**

In vitro BrdU proliferation and Annexin V cell death analyses were performed on PANC-1 and MIA PaCa-2 PDAC cell lines exposed to conditioned medium of different macrophage subsets. Macrophage secreted factors were measured by transcript analysis and ELISA. Therapeutic antibodies were used to functionally establish the impact of the identified cytokine on PDAC proliferation.

**Results:**

Proliferation and cell death assays revealed that only M0 macrophages harbor anti-tumorigenic activities and that M1, M2, and TAMs do not. mRNA analysis and ELISA results suggested TNF-α as a potential candidate to mediate M0 macrophage induced cell death. To demonstrate the importance of TNF-α in M0 macrophage-induced cell death, PANC-1 and MIA PaCa-2 cell-lines were exposed to M0 macrophage conditioned medium in the presence of the TNF-α inhibitor Infliximab, which effectively diminished the anti-tumor activities of M0 macrophages.

**Conclusion:**

Newly tumor-infiltrated naive M0 macrophages exert anti-tumorigenic activities via TNF-α secretion. Their subsequent differentiation into either M1, M2, or TAM subsets reduces TNF-α levels, thereby abolishing their cytotoxic activity on PDAC cells. These data suggest that reestablishing TNF-α secretion in differentiated macrophages might yield a therapeutic benefit.

**Supplementary Information:**

The online version contains supplementary material available at 10.1186/s12885-020-07697-1.

## Background

Pancreatic ductal adenocarcinoma (PDAC), the most common form of pancreatic cancer, is a highly malignant disease with very poor reported 5-year survival rates of less than 8% [1]. Despite numerous research efforts and the development of therapeutics to overcome the low overall survival in PDAC, disease progression rates and mortality have declined only marginally [2]. High therapy resistance and complex interactions with the immune system are some of the major features that limit the efficacy of current treatments against PDAC [3].

As part of the innate immune system, monocytes are recruited to damaged tissue as the initial response and are subsequently activated to become macrophages. These newly infiltrated macrophages, also known as naïve (M0) macrophages, then respond to extracellular signals in the tissue and differentiate into either classically activated macrophages (M1, also known as pro-inflammatory) or alternatively activated macrophages (M2, also known as anti-inflammatory) [4]. However, in tumor tissue, infiltrated macrophages can differentiate into a specific subset of macrophages known as tumor-associated macrophages (TAMs), which often harbor M2-like characteristics [5]. Macrophage infiltration in solid tumors is common, and despite being part of an immune response, it is often associated with poor prognosis and metastatic disease [6].

In pancreatic cancer, the role of TAMs in metastasis and chemoresistance has been studied extensively. TAMs are generally considered to be tumor-promoting members of the PDAC tumor microenvironment, which should be targeted to limit disease progression [7]. Notwithstanding, when and how the initial anti-tumor macrophage response transitions to a tumor-promoting macrophage phenotype are unclear. Given the increasingly recognized importance of the immune system in cancer and its potential for therapy development, the identification of anti-tumor macrophage responses could be an essential step forward to harness the immune responses at play in PDAC. This study demonstrates how naïve M0 macrophages differ from further differentiated macrophages in terms of anti-tumorigenic activities. We further identify M0 macrophage secreted c as a potent tumor cell-death inducer.

## Methods

### Cell-lines and cell culture

Human PANC-1 and MIA PaCa2 pancreatic cancer cells (ATCC, Manassas, VA) were cultured in DMEM (Gibco, Thermo Fischer Scientific, Waltham, MA) with 4.5 g/mL glucose, and human THP-1 cells (ATCC) were cultured in RPMI-1640 (Gibco). All cell culture media were supplemented with 10% fetal bovine serum (FBS), L-glutamine (2 mM), except RPMI-1640 media for conditioned media experiments, which was supplemented with 1X GlutaMAX (Gibco), penicillin (100 units/mL) (Gibco), and streptomycin (500 μg/mL) (Lonza, Basel, Switzerland) in regular cell culture procedures. Cultures were incubated in 5% CO_2_ incubators at 37 °C. All cell lines were authenticated by STR profiling (Promega PowerPlex, Leiden, Netherlands) and tested for mycoplasma by PCR monthly.

### Generation of M0, M1, M2, and TAM macrophages from THP-1 cells

**Fig. 1:**
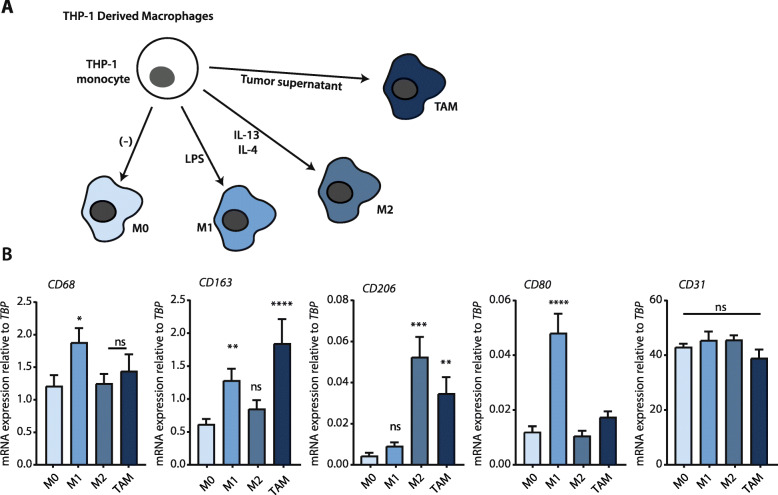
Macrophage subsets used in this study. **a** Schematic representation of the macrophage differentiation procedure and the resulting macrophage subtypes. **b** Relative mRNA expression of CD68, CD163, CD206, CD80, and CD31 on the macrophage subtypes. On the figure shown is the mean ± SEM (*n* = 6); One-way ANOVA was used to assess statistical significance. Relative expression levels were calculated using the dCt method and normalized to the expression of the reference gene *TBP*

THP-1 cells were initially treated with 150 nM phorbol 12-myristate 13-acetate (PMA, Sigma, St. Louis, MO, USA) for 24 h in RPMI-1640 medium. Next, activated THP-1 cells were washed with fresh medium to remove PMA. Cells were cultured in refreshed, fully supplemented RPMI-1640 medium for another 24 h, after which the medium was renewed once more. Without any other additions, macrophages were considered M0 macrophages at this stage (see Fig. 1 for validation). M0 macrophages were treated with 1 ng/ml LPS (Ultrapure, Invivogen, Toulouse, France) for 24 h to generate M1 macrophages. M0 macrophages were treated with 20 ng/ml recombinant IL-4 (Peprotech, Rocky Hill, NJ) and IL-13 (Peprotech) each for 72 h to generate M2 macrophages. Finally, for the generation of tumor-associated macrophages (TAM), M0 macrophages were treated with a supernatant mix (collected from PANC-1 and MIA PaCa-2 cells at ~ 80% confluence) in a 1:1 dilution with fresh complete RPMI-1640 media for 72 h. At the end of each incubation, macrophages were washed twice with a fully supplemented RPMI-1640 medium to remove the cytokines and other factors from the flask. Media collections were performed after 48 h of incubation. Collected media centrifuged at 1200 rpm for 4 min to remove cell debris, filtered using 0.2 μm syringe filters (Corning, Corning, New York), and stored at 4 °C. For experimental procedures, all conditioned media were diluted 1:1 with fresh media (DMEM) to ascertain the medium’s appropriate nutrient content.

### Generation of monocyte-derived macrophages from peripheral blood mononuclear cells

Peripheral blood mononuclear cells (PBMC) were obtained by Ficoll-Paque (GE Healthcare, Chicago, IL, USA) density centrifugation from whole blood collected from a single healthy volunteer (following institutional standard operating protocol and under the approval of Medical Ethics Review Committee) in lithium-heparin coated blood tubes (BD, Franklin Lakes, NJ). For monocyte isolation from PBMC, the MACS Monocyte Isolation kit with CD14+ magnetic beads was used with MACS LS Columns according to the manufacturer’s manual (Miltenyi Biotec, Bergisch Gladbach, Germany). Isolated monocytes were plated in fully supplemented RPMI media and treated with either 50 ng/ml M-CSF (Prospec, Rehovot, Israel) or 50 ng/ml GM-CSF (Prospec) for 6 days to obtain M0 subtype monocyte-derived macrophages (MDM); media were refreshed together with the cytokines every 2 days. To obtain the M1 and M2 MDM subtypes, all media were removed on day six, after which the M0 macrophages were treated for 24 h with 50 ng/ml IFN-γ (Peprotech) on the previously GM-CSF treated group or with 20 ng/ml IL-4 (Peprotech) and IL-13 (Peprotech) on the previously M-CSF treated group to obtain M1 and M2 MDMs respectively. Conditioned media from all subtypes were collected and processed as for the THP-1 derived macrophage media.

### Quantitative real-time PCR

Total RNA of the samples was isolated using a NucleoSpin RNA miniprep kit with DNase treatment (Macherey Nagel, Düren, Germany), and cDNA from the samples were synthesized from total RNA (with 1000 ng total RNA per sample) using M-MLV-RT (Promega, Leiden, Netherlands) and random hexamers (Qiagen, Hilden, Germany). Quantitative RT-PCR was performed to determine the mRNA levels, with Sensifast SYBR No-Rox Kit (Bioline, London, UK), measured on LightCycler 480 II (Roche, Basel, Switzerland). Relative expression levels were calculated using the comparative threshold cycle (dCt) method and further normalized to expression of the reference gene TBP. All primer sequences of the analyzed genes are shown in Supplementary Table 1.

### BrdU proliferation assay

PANC-1 and MIA PaCa2 cells were seeded in black, clear-bottom 96-well plates (Corning) in DMEM without serum. The following day, macrophage conditioned media were added in a 1:1 dilution with complete DMEM. BrdU labeling solution was given to cells after 48 h of conditioned media treatment. All experimental steps in the BrdU assay were performed according to the manufacturer’s instructions (Chemiluminescent BrdU assay, Roche). Luminescence was measured on a Synergy HT Biotek Microplate Reader (Biotek Instruments, Winooski, VT). For testing the functional importance of TNF-α in M0 conditioned medium, we included human recombinant TNF-α (Sigma) in increasing concentrations from 10 pg to 10 ng and the TNF-α inhibitor Infliximab (Remicade, MSD, Kenilworth, NJ) at 25 μg/ml.

### TNF-α ELISA

A commercially available ELISA kit (R&D Systems, Minneapolis, MN) was used to determine TNF-α levels from THP-1- and MDM-derived macrophage supernatants. Samples were measured in 4 biological replicates. All experimental steps were performed according to the manufacturer’s instructions, and the absorbance was measured with the Synergy HT Biotek microplate reader (Biotek Instruments) at 450 and 655 nm.

### Flow cytometry detection of Annexin V

Cells were treated for 48 h with different macrophage supernatants and with a 1:1 DMEM-RPMI mix as control. Cell supernatants were collected in a tube to include non-adherent cells. Cells were detached using trypsin, and this cell fraction was pooled with the cell supernatant in a 15 mL tube. The mix was pelleted by centrifugation at 1200 rpm for 5 min. The pellet was re-suspended in Annexin V binding buffer (BD) and distributed to 96-well plates for staining. Each well contained 100 μl of cell mix and 1 μl Annexin V FITC (BD). The plate was incubated on ice, in the dark, for 1 h. After incubation, the plate was washed twice with Annexin V binding buffer, and cells were re-suspended in 200 μL in the same buffer for measuring. The measurements were performed on a FACS Canto II (BD). Data were analyzed using FLOWJO v10 (FlowJo LLC, Ashland, OR). Cells were gated initially based on FCS and SSC for the main cell population, and then on FCS-H and FCS-W to obtain single cells. FITC-positive populations were gated based on isotype antibody control samples. For quantification, Geometric Mean Fluorescent Intensity (gMFI) on FITC values were used.

### Statistical analysis

Data were presented as mean ± SEM. Statistical analyses were performed using GraphPad PRISM 7.0 (Graphpad Software Inc., La Jolla, CA). Differences were considered statistically significant at a *p*-value of less than 0.05. For further details of the statistical analysis, see figure legends. *P*-values on graphs are indicated by asterisks with * *p* < 0.05, ** *p* < 0.01, *** *p* < 0.001, and **** *p* < 0.0001.

## Results

### Anti-tumor activity of macrophages is restricted to the M0 (naïve) subset

The crosstalk between newly infiltrated monocytes and other constituents of the cancer microenvironment (most notably tumor cells) leads to the differentiation of monocytes into macrophages. Subsequent tumor stimuli then induce differentiation of these macrophages into TAMs, which exhibit pro-tumorigenic properties [8]. Recent work from our group has shown that M0 (naïve) macrophages harbor cytotoxic properties against PDAC cell lines [9], but how this relates to the tumor-promoting capacities of other macrophage subsets remains unclear. We hypothesized that, as macrophages differentiate in the tumor microenvironment, their anti-tumor activities would gradually be lost, with TAMs bearing no anti-tumor activity at all. To test this hypothesis, we turned to an isogenic system to generate the four major subtypes of macrophages; M0 (PMA induced THP-1 monocytes; Fig. 1a), M1, M2, and TAM. Macrophage differentiation was successful, as evident from mRNA analysis of the specific markers CD68 (for all macrophages), CD163 (predominantly for TAM), CD206 (for TAM and M2), CD80 (for M1), and CD31 (to confirm monocytic lineage) (Fig. 1b).

**Fig. 2:**
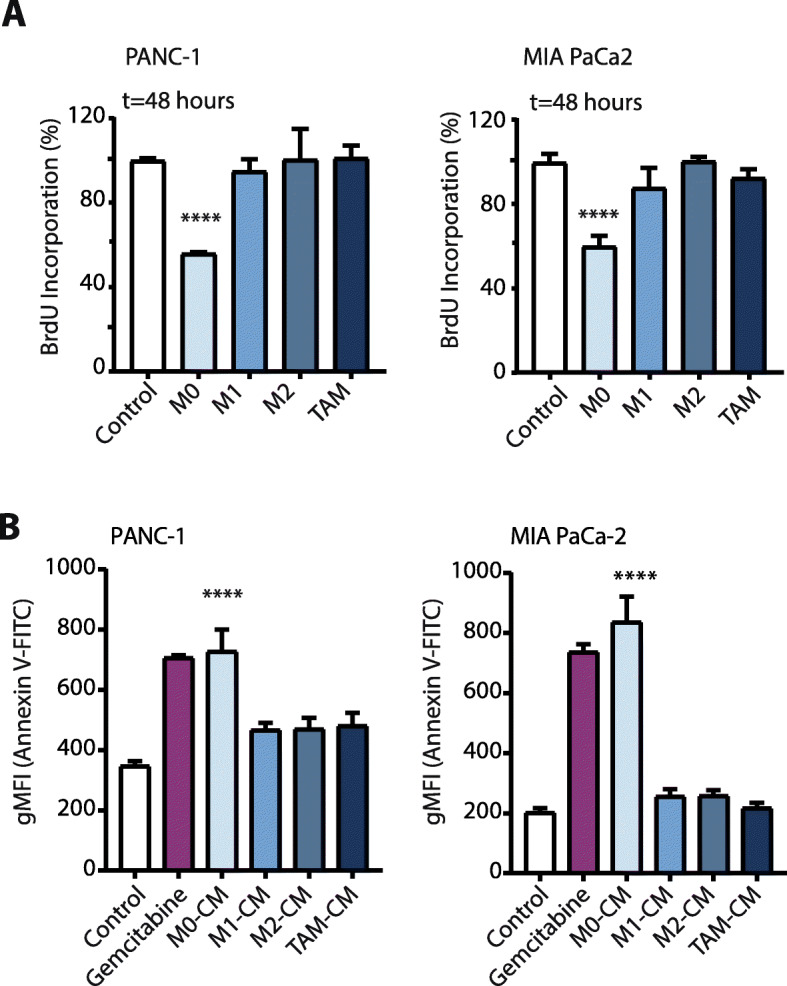
Anti-tumorigenic activities on PDAC cells are unique to the M0 subset of macrophages. **a** BrdU proliferation assay of PANC-1 and MIA PaCa-2 cells in RPMI-1640 (negative-control) or conditioned media from different macrophage subtypes at t = 48 h. On the figure shown is the mean ± SEM (*n* = 4); One-way ANOVA was used to assess statistical significance. **b** Annexin V-FITC positive cells in negative-control (RPMI-1640), conditioned media from different macrophage subtypes, or Gemcitabine (as positive-control) treated PANC-1 and MIA PaCa-2 cells at t = 48 h. Plotted is the Geometric Mean Fluorescence Intensity (gMFI) on the FITC channel (Annexin V density). On the figure shown is the mean ± SEM (*n* = 3); One-way ANOVA was used to assess statistical significance

To assess the impact of these different macrophage subtypes on cancer cell viability, we cultured PANC-1 and MIA PaCa-2 PDAC cell lines with conditioned media from the different macrophage subsets. Proliferation was measured by BrdU incorporation. We found that both cell lines treated with M0-conditioned media (M0-CM) had significantly fewer proliferating cells when compared to control, and, of note, compared to all other macrophage media (Fig. 2a). To test whether this observation was due to M0-CM-induced cell cycle arrest or cell death, we stained the macrophage media-treated cells with a fluorescently labeled Annexin V and measured its levels by flow cytometry. This revealed that M0-CM treated cells had markedly increased Annexin V positivity (Fig. 2b), indicating that M0 macrophages induce apoptosis in PDAC cells, whereas more differentiated macrophage subsets do not.

### M0 macrophage secreted TNF-α is essential for anti-tumorigenic activities

**Fig. 3:**
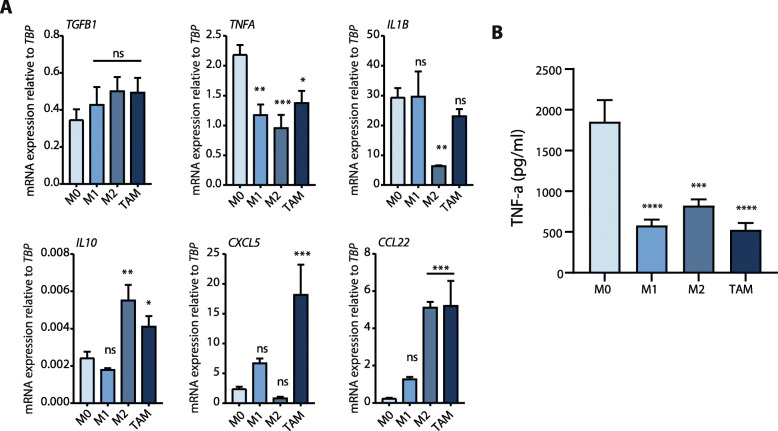
M0 macrophages express higher levels of TNF-α. **a** Relative mRNA expression of TGF-β, TNF-α, IL-1β, IL10, CXCL5, and CCL22 in the indicated macrophage subsets. On the figure shown is the mean ± SEM (*n* = 4); One-way ANOVA was used to assess statistical significance. Relative expression levels were calculated using the dCt method and normalized to the expression of *TBP*. **b** TNF-α levels in conditioned media from all given macrophage subsets, measured by ELISA. On the figure shown is the mean ± SEM (*n* = 4); One-way ANOVA was used to assess statistical significance

Next, we investigated potential key cytokines secreted by the macrophage subsets. We included both anti- and pro-inflammatory cytokines involved in tumor suppression such as TNF-α, IL-1β, and tumor promotion such as IL-10, CCL22, CXCL5, and TGF-β (selection of cytokines based on ref. (10, 11)). Since only M0 macrophages exerted anti-tumor effects, we looked for a significantly different expression of any cytokine in this particular macrophage subset. mRNA analysis of selected cytokines revealed a marked increased expression of TNFA in the M0 group (Fig. 3a), whereas other cytokines exhibited either no significant differences or non-M0 specific expression. ELISA measurements of TNF-α in macrophage media further confirmed that M0 macrophages secrete significantly more TNF-α than do other macrophage subsets (Fig. 3b).

**Fig. 4:**
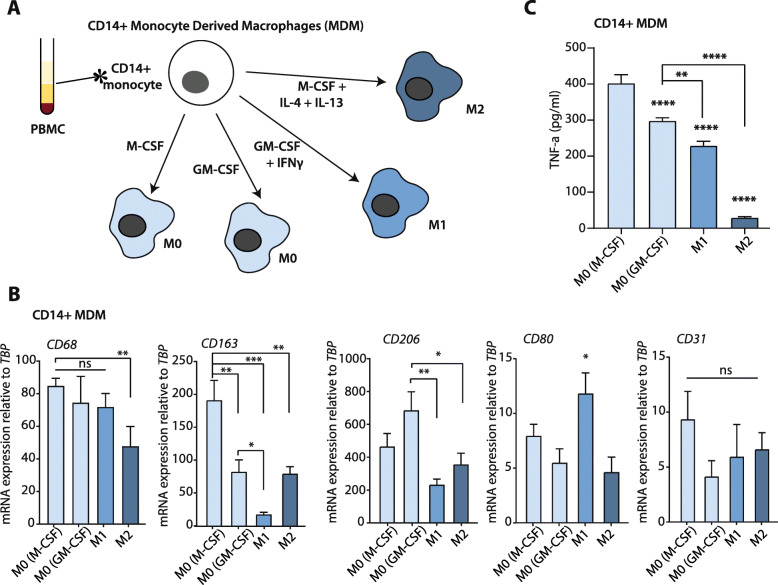
Isolation and TNF-α expression of CD14+ monocyte-derived macrophages (MDM). **a** Schematic representation of the macrophage differentiation procedure and the resulting macrophage subtypes. **b** Relative mRNA expression of *CD68, CD163, CD206, CD80*, and *CD31* on the macrophage subtypes. On the figure shown is the mean ± SEM (*n* = 4); One-way ANOVA was used to assess statistical significance. Relative expression levels were calculated using the dCt method and normalized to the expression of the reference gene *TBP*. **c** TNF-α levels in conditioned media from all different macrophage subsets, measured by ELISA. On the figure, shown is the mean ± SEM (*n* = 4); One-way ANOVA was used to assess statistical significance

The THP-1 cell line is a human monocytic cell line derived from an acute myeloid leukemia (AML) patient. Therefore, due to its cancerous nature, the cell line may exhibit differences from healthy monocytes. We aimed to confirm the role of TNF-α secretion by M0 macrophages derived from monocyte-derived macrophages (MDMs) isolated from peripheral blood mononuclear cells (PBMC). CD14+ monocytes were isolated from PBMC and further processed to yield M0 (M-CSF primed or GM-CSF primed), M1, and M2 subtypes (Fig. 4a). To confirm the differentiation, phenotype analysis was done by mRNA analysis of specific surface markers (as for Fig. 1); CD68, CD163, CD206, CD80, and CD31 (Fig. 4b). As expected, all macrophage subtypes had similar CD69 and CD31 expression, whereas CD80 was only enhanced in M1 macrophages. Importantly, similar to THP-1-derived M0 macrophages, M-CSF and GM-CSF primed MDM M0 macrophages have significantly enhanced TNF-α secretion compared to MDM-derived M1 and M2 macrophages (Fig. 4c).

**Fig. 5:**
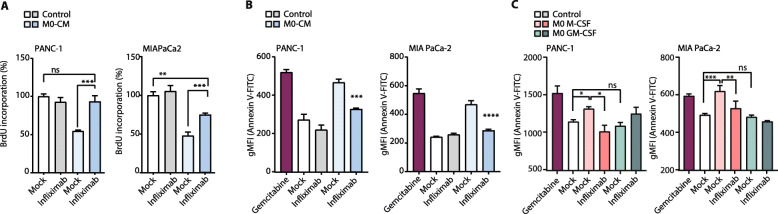
Infliximab inhibits the cytotoxic activity of M0 macrophages. **a** BrdU proliferation assay of PANC-1 and MIA PaCa-2 cells in RPMI-1640 (negative-control) or M0-CM with 25 μg/ml Infliximab where indicated. On the figure shown is the mean ± SEM (*n* = 4); One-way ANOVA was used to assess statistical significance. **b** Annexin V-FITC positive cells in control, M0-CM with or without 25 μg/ml Infliximab where indicated, or Gemcitabine (as positive control) treated PANC-1 and MIA PaCa-2 cells at t = 48 h. Plotted is the Geometric Mean Fluorescent Intensity (gMFI) on the FITC channel (Annexin V density). On the figure shown is the mean ± SEM (*n* = 3); One-way ANOVA was used to assess the significance. **c** Annexin V-FITC positive cells in control, M0 M-CSF, or M0 GM-CSF with or without 25 μg/ml Infliximab where indicated, or Gemcitabine (as positive-control) treated PANC-1 and MIA PaCa-2 cells at t = 48 h. Plotted is the Geometric Mean Fluorescent Intensity (gMFI) on the FITC channel (Annexin V density). On the figure shown is the mean ± SEM (*n* = 3); One-way ANOVA was used to assess the significance

Titration curves with recombinant TNF-α show that TNF-α levels in the range as secreted by M0 macrophages induces cell death of PANC-1 cells (Sup. Figure S1). Following these findings, which strongly point to TNF-α as a critical cytokine in early-macrophage induced tumor cell death, the question arises whether TNF-α inhibition can reverse the M0 induced cell death. To test his, we used Infliximab, a TNF-α inhibitor in clinical use against ulcerative colitis and Crohn’s disease [12]. Infliximab treatment almost completely diminished the growth-inhibitory effects of THP-1-derived M0-CM on PANC-1 and MIA PaCa-2 cell-lines (Fig. 5a). Likewise, treatment with Infliximab prevented THP-1-derived M0-CM induced apoptosis of both PDAC cell lines (Fig. 5b). Interestingly, CM of M-CSF primed MDM-derived M0 macrophages also induced apoptosis of PDAC cells in a TNF-α dependent manner (Fig. 5c). Altogether these findings suggest that M0 macrophage-secreted TNF-α is a robust anti-tumor cytokine, but that exposure of macrophages to tumor microenvironmental cues functions to convert macrophages into subsets that no longer express sufficient TNF-α to exert anti-tumor activities.

## Discussion

Increased inflammation and enhanced macrophage influx in cancer tissue are associated with poor prognosis and increased risk of developing therapy resistance and metastasis [13]. Numerous studies in murine PDAC models have shown that macrophage influx into the tumor is accompanied by gemcitabine resistance and metastatic burden [14–16]. Despite these tumor-promoting effects [17–19], our group has previously shown that early macrophage infiltrates instead initially exert cytotoxic effects on PDAC cells. However, factors secreted by infiltrated macrophages, such as MMP9, induce epithelial to mesenchymal transition (EMT) of PDAC cells [9]. This EMT enables PDAC cells to escape cytotoxicity, also that induced by macrophages, further underscoring the dual contributions of macrophages to tumor progression [9]. An abundance of studies has reported tumor-associated macrophages (TAMs; M2-like differentiated macrophages) to be associated with increased tumor growth and therapy resistance [20], which has led to the notion that macrophage entry into the tumor should be prevented. However, clinical trials aimed to block monocyte recruitment to reduce TAM content did not yield promising results [21]. Consequently, the majority of subsequent trials have focused on reprogramming TAMs into anti-tumorigenic macrophages [21]. Our results suggest that this latter approach is most promising, as early infiltrated macrophages exert strong anti-tumor effects, and their influx should not be hampered. Ideally, the differentiation of naïve M0 macrophages should be blocked. However, a complicating factor for the development of therapies that target monocyte infiltration into the PDAC tumor tissue (or prevent early differentiation events) is that at the moment of diagnosis, the tumor microenvironment is at least several years old, and the window of opportunity to retain or boost M0 activity is likely lost.

Although the immune regulation in the tumor microenvironment is a complex phenomenon, it is known that tumor cells induce the downregulation of pro-inflammatory cytokines during TAM differentiation [22]. Bearing in mind that, M0 macrophages to be naïve macrophages that have not been exposed to any tumor tissue (unlike the other more differentiated macrophage subsets), this study supports the notion that the tumor environment actively instructs macrophages to be less detrimental to tumor growth. We propose that it is unlikely that the complex mixture of cues from the tumor microenvironment that instruct macrophages can be adequately targeted, and instead posit that the macrophage-intrinsic mechanisms that suppress TNF-α secretion should be studied. This could preserve macrophage-mediated cell-death in the tumor. For instance, IL-1 Receptor Associated Kinase-M (IRAK-M) activity is known to be upregulated in tumor-exposed macrophages with significantly high expression in TAMs, which in return, suppresses TNF-α secretion [23]. Reprogramming TAMs to secrete increased amounts TNF-α for instance, by exposure to (yet unknown) ligands, could be an interesting avenue to pursue.

We would like to point out several limitations of our study. In part, these are technical in nature; a publicly available lymphoma-derived monocyte cell line (THP-1), and single-donor PBMC isolated monocyte-derived macrophages (MDM) as a primary monocyte cell culture, were used as in vitro proxies for macrophage subsets. One of the technical limitations of using healthy primary monocytes is that obtaining tumor-associated macrophages (TAM) is not possible. Moreover, although M-CSF and GM-CSF treated MDMs may be considered as naïve M0 macrophages, they are already primed towards M2 (M-CSF) or M1 (GM-CSF) subtypes [24]. Indeed, both cell-surface markers (Fig. 4 and [24]) and TNF-α secretion suggest that especially the GM-CSF-primed MDM-derived M0 macrophages are less naïve as THP-1 derived M0 macrophages. In line with this notion, M-CSF-primed MDM-derived M0 macrophages mimic the results of THP-1-derived M0 macrophages, whereas GM-CSF-primed MDMs do not. Most likely, this is due to the fact that GM-CSF-primed MDMs secrete less TNF-α as compared to M-CSF-primed MDM-derived M0 macrophages (Fig. 4c). Another limitation of our study is that no in vivo experiments were performed to determine the contributions of M0 macrophages to tumor growth and the effects of TNF-α inhibition in such a setting. Finally, an issue that urges clarification in future studies is the discrepancy between the large difference in cell killing by M0 macrophages and the complete reliance on TNF-α on the one hand and the relatively modest difference in TNF-α levels in the macrophage subsets found by ELISA on the other. This suggests that other cytokines can be at play but that TNF-α levels determine how the balance of the tumor-promoting and –limiting contributions of these cytokines add up.

A more fundamental question arises from the efficacy with which Infliximab appears to inhibit macrophage-mediated cytotoxicity. If indeed this antibody prevents the tumor inhibitory effects of early macrophage infiltrates in cancers (amongst which PDAC), one would expect this to have become apparent from clinical data and longitudinal studies following the approval of this drug 20 years ago. One argument to explain this discrepant notion is that for PDAC, it is now recognized that the progress from premalignant cell-of-origin to full-blown carcinoma takes several decades [25]. This implies that any tumor-promoting effects of TNF-α inhibition may become apparent from epidemiological analyses in the years to come.

## Conclusion

Early macrophage infiltrates in the tumor microenvironment are intrinsically anti-tumorigenic, but this is quickly thwarted by differentiation into TAMs that harbor pro-tumorigenic activities. The current therapeutic focus has been to block macrophage entry and thereby TAM content. In this study, we identify TNF-α as a potent cytokine in early macrophages, which is downregulated following further macrophage differentiation. Altogether these findings suggest that restoring TNF-α expression in TAMs might reverse their pro-tumorigenic potential and that this could be a more promising strategy than preventing macrophage influx.

## Supplementary Information

**Additional file 1: Table S1.** Primer sequences for qPCR analysis of mRNA expression.

**Additional file 2: Figure S1.** Cytotoxicity of human recombinant TNF-α on PANC-1 cell-line is dose-dependent. PANC-1 cells were treated with human recombinant TNF-α in increasing concentrations (10 ng to 10 pg). Annexin V-FITC positive cells in control media (black dashed line given as baseline), or Gemcitabine (as the positive control, purple dash bar given as cell-death comparison) treated PANC-1 cells at t = 48 h. Plotted is the Geometric Mean Fluorescent Intensity (gMFI) on the FITC channel (Annexin V density), and decreasing concentration is plotted on Y-axis. On the figure shown is the mean ± SEM (*n* = 2).

## Data Availability

Research materials are available on request.

## Abbreviations

TNF-α: Tumor necrosis factor-α
PDAC: Pancreatic ductal adenocarcinoma
MDM: Monocyte-derived macrophages
PBMC: Peripheral blood mononuclear cells
TAM: Tumor-associated macrophages
IL: Interleukin
CCL22: C-C motif chemokine 22
CXCL5: C-X-C motif chemokine 5
TGF-β: Transforming growth factor-β
PMA: Phorbol 12-myristate 13-acetate
EMT: Epithelial-to-mesenchymal transition
M-MLV-RT: Moloney murine leukemia virus reverse-transcriptase
ELISA: Enzyme-linked immunosorbent assay
RT-PCR: Reverse-transcriptase polymerase chain reaction
CM: Conditioned medium
cDNA: Complementary DNA
