# Supplementary material for: Early macrophage infiltrates impair pancreatic cancer cell growth by TNF-α secretion
Source: BMC Cancer. 2020 Dec 2;20:1183. doi: 10.1186/s12885-020-07697-1 (PMC7709323; doi:10.1186/s12885-020-07697-1)
Supplement: Supplementary file 1 — Additional file 1: Table S1. Primer sequences for qPCR analysis of mRNA expression. [file 12885_2020_7697_MOESM1_ESM.docx]

| Gene | Forward | Reverse |
| --- | --- | --- |
| *hCCL22* | CCCCAGTCACCTGCTGTTAT | TCCTGAACCCACTTCTGCTT |
| *hCD163* | GAGTCAGCTCTTTGGGATTGC | CCAAATTGGATCCATCTGAGC |
| *hCD206* | GCGTGGCTGCAGATGGAAACATCTA | TGTACCTCACCCTCCACTTATCAGTCCA |
| *hCD68* | ATTCATGCAGGACCTCCAGCA | ATGTCCACTGTGCTGCGTG |
| *hCXCL5* | CTCCAAGGTGGAAGTGGTAGC | TCCTTGTTTCCACCGTCCAA |
| *hIL10* | GATCCAGTTTTACCTGGAGGAG | TTAAAGGCATTCTTCACCTGCT |
| *hIL-1b* | AAAAAGCTTGGTGATGTCTGGT | CGCAGGACAGGTACAGATTCTT |
| *hTBP* | ATCCCAAGCGGTTTGCTGC | ACTGTTCTTCACTCTTGGCTC |
| *hTGF-β* | CGTGGAGGGGAAATTGAGG | GTAGTGAACCCGTTGATGTCCACT |
| *hTNF-α* | CAGGGACCTCTCTCTAATCAGC | GCTGGTTATCTCTCAGCTCCAC |
| *hCD31* | GACCCTTCTGCTCTGTTCA | CTGAGGCTTGACGTGAGAG |
| *hCD80* | GGGAAATGTCGCCTCTCTGA | GTGGATTTAGTTTCACAGCTTG |

**Supplementary Table 1.** Primer sequences for qPCR analysis of mRNA expression.
